# Cryogenic TEM imaging of artificial light harvesting complexes outside equilibrium

**DOI:** 10.1038/s41598-022-09496-z

**Published:** 2022-04-01

**Authors:** Sundar Raj Krishnaswamy, Ivo A. Gabrovski, Ilias Patmanidis, Marc C. A. Stuart, Alex H. de Vries, Maxim S. Pshenichnikov

**Affiliations:** 1 Zernike Institute for Advanced Materials, University of Groningen, Nijenborgh 4, 9747 AG Groningen, the Netherlands; 2 Groningen Biomolecular Sciences and Biotechnology Institute, University of Groningen, Nijenborgh 7, 9747 AG Groningen, the Netherlands

**Keywords:** Self-assembly, Imaging techniques

## Abstract

The energy transport in natural light-harvesting complexes can be explored in laboratory conditions via self-assembled supramolecular structures. One such structure arises from the amphiphilic dye C8S3 molecules, which self-assemble in an aqueous medium to a double-wall cylindrical nanotube reminiscent of natural light-harvesting complexes found in green sulphur bacteria. In this paper, we report a way to investigate the structure of inner nanotubes (NTs) alone by dissolving the outer NTs in a microfluidic setting. The resulting thermodynamically unstable system was rapidly frozen, preventing the reassembly of the outer NT from the dissolved molecules, and imaged using cryogenic transmission electron microscopy (cryo-TEM). The experimental cryo-TEM images and the molecular structure were compared by simulating high-resolution TEM images, which were based on the molecular modelling of C8S3 NTs. We found that the inner NT with outer walls removed during the flash-dilution process had a similar size to the parent double-walled NTs. Moreover, no structural inhomogeneity was observed in the inner NT after flash-dilution. This opens up exciting possibilities for functionalisation of inner NTs before the reassembly of the outer NT occurs, which can be broadly extended to modify the intra-architecture of other self-assembled nanostructures.

## Introduction

**Figure 1:**
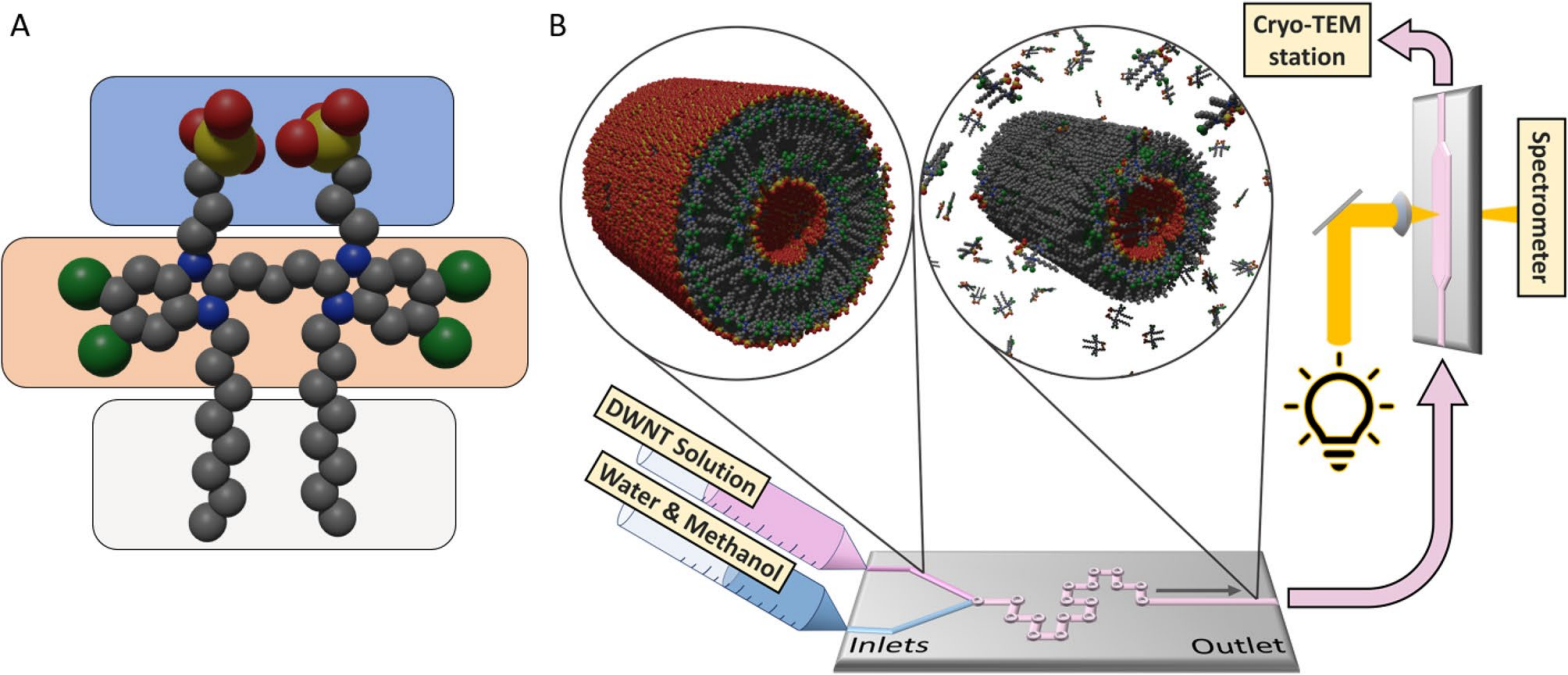
Schematic of the experimental setup for the flash-dilution of C8S3 DWNTs. (**A**) Structure of the amphiphilic C8S3 molecule (red: oxygen, yellow : sulphur, green: chlorine, grey: carbon, blue: nitrogen) where different functional entities are highlighted (blue—hydrophilic group, orange—chromophore, grey—hydrophobic group). (**B**) The two syringe pumps supply the DWNT solution and methanol–water mixture to the microfluidic tear drop mixer, where the flash-dilution process is carried out in a controlled fashion. The outlet of the mixer is short-connected to a microfluidic flow cell, where the absorption spectrum of the flash-diluted NTs is continuously monitored. The flash-diluted NTs are collected at the end of the flow cell, rapidly frozen in the cryo-TEM station and imaged using cryo-TEM. The insets show a visual representation (rendered using Blender) of DWNTs and flash-diluted NTs, along with dissolved molecules; for simplicity, the solvent molecules are not shown.

In nature, light-harvesting complexes with strong intermolecular couplings play a vital role in the process of photosynthesis, by facilitating the transport of excitation energy to the reaction centres^1,2^. To better understand the energy transport processes, the natural systems have been extensively modelled by simpler and more controllable artificial systems^3,4^. Among these, double-walled nanotubes, (DWNTs) which self-assemble from amphiphilic C8S3 molecules (Fig. 1A) in an aqueous environment^5,6^, are especially interesting as they bear strong structural similarity to chlorosomes found in green sulphur bacteria^7–9^, which are optimised for photosynthesis in poorly lit environments^8^. It is generally agreed^6,10,11^ that the C8S3 DWNT system is composed of strongly coupled chromophores organised in inner and outer supramolecular cylindrical structures (Fig. 1B, left inset), which has a significant influence on the optical properties of the system.

Strong coupling among the chromophores leads to the formation of delocalised excitations, the excitons^10,12^. These travel back and forth along the nanotubes (NTs), but could also migrate from one NT to another^11^. To prevent such cross-talk between the NTs, the inner NT can be decoupled from the DWNT structure by a process called flash-dilution^10,13^, i.e. rapid mixing of the DWNT aqueous solution with a mixture of methanol and water. This allows the selective dissolution of the outer NT (Fig. 1B, right inset), as was concluded based on the disappearance of the excitonic absorption peak associated with the outer NT^6,11^. Modelling the optical properties of the inner NT requires knowledge of its structure, but structural studies (e.g. cryo-TEM) are limited by rapid reassembly (within a few minutes^10^) of the dissolved out-of-equilibrium outer NT molecules.

Here, we solved this problem by using a combination of microfluidics and cryo-TEM (Fig. 1B) to image the isolated inner NT. Cryo-TEM imaging was performed on the inner NTs, which were rapidly frozen ∼40 s after microfluidic flash-dilution, thus preventing the reassembly process. The experimental cryo-TEM images were compared to the ones calculated using high-resolution transmission electron microscopy (HRTEM) software^14^ with the input from molecular modelling of C8S3 NTs. We found that the inner NTs were largely unaffected by flash-dilution as compared to their DWNT counterparts. Furthermore, the resulting ensemble of inner NTs was as structurally homogeneous as the parent DWNTs, despite the potential destructiveness of flash-dilution. These findings open up exciting possibilities for controlled modifications of the intra-architecture of self-assembled nanostructures, which would alter the optical properties of the system.

## Results and discussion

### Microfluidic flash dilution

**Figure 2:**
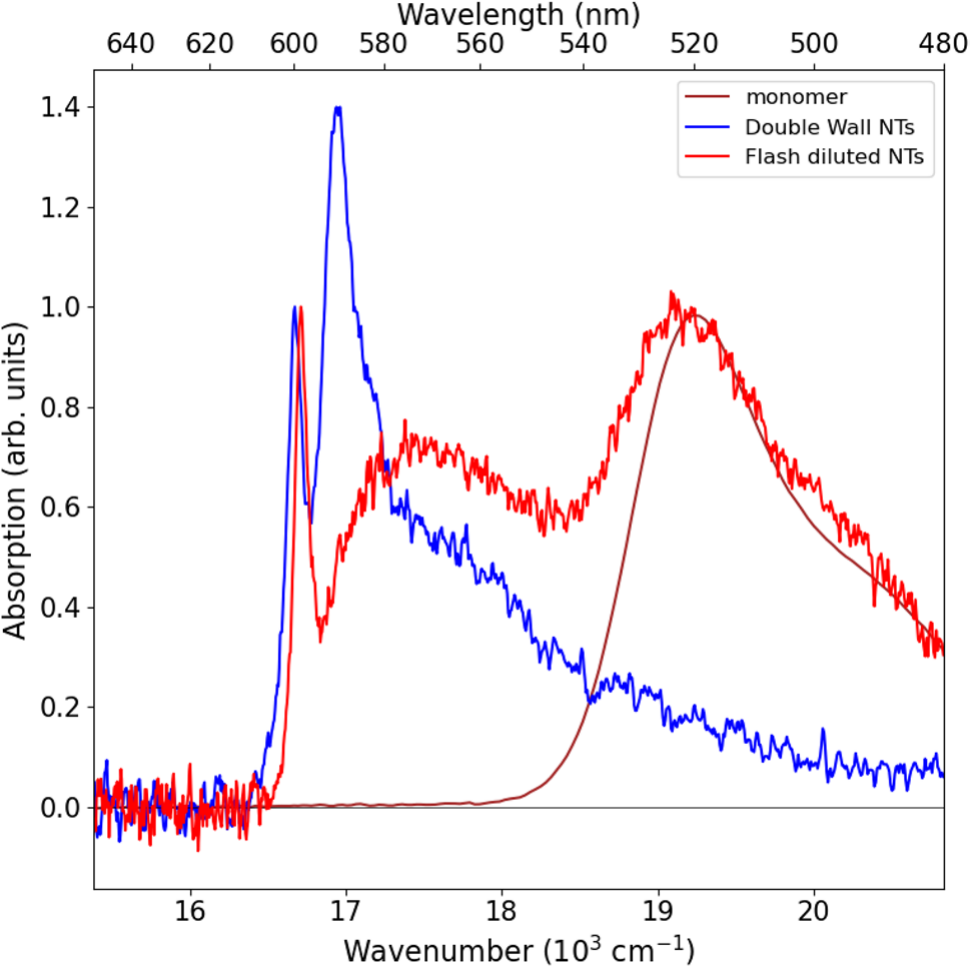
Absorption spectra of C8S3 monomers dissolved in methanol (brown), DWNTs (blue) and flash-diluted NTs (red). For the purpose of comparison, the spectra of DWNTs and flash-diluted NTs are normalised to the amplitude of the peak at ∼600 nm, while the monomer spectrum is normalised to the amplitude of the peak at ∼520 nm. Note a ∼40 cm^−1^ blue shift of the 600 nm peak of flash-diluted NTs with respect to the spectrum of DWNTs.

Addition of water to a stock solution of C8S3 dye molecules dissolved in methanol (see “Materials and methods”) results in the formation of DWNTs^10,15^, which is spectroscopically observed by a strong red shift of ∼70 nm (∼2300 cm^−1^) in the absorption spectrum (Fig. 2, blue spectrum). Two narrow peaks at ∼600 nm (∼16,673 cm^−1^) and ∼590 nm (∼16,963 cm^−1^) correspond to excitonic transitions in the inner and outer NTs, respectively^16,17^. The shoulder below 580 nm is attributed to higher-laying excitonic transitions^16^.

The process of flash-dilution is carried out by mixing DWNT solution with a 1:1 (v/v) mixture of methanol and water in a microfluidic teardrop mixer^18^. The disappearance of the peak at ∼590 nm has been previously attributed to the dissolution of the outer NT^10,18^. A broad peak at ∼574 nm corresponds to higher-energy excitonic transitions^10,16^; the contribution from bundles can be ruled out as they were hardly observed in cryo-TEM images of the DWNT solution (Supplementary Information I). Moreover, the ∼604 nm absorption peak characteristic for the bundles^5,19^ was hardly observed (Supplementary Information I).

### Cryo-TEM imaging of DWNTs and flash-diluted NTs

**Figure 3:**
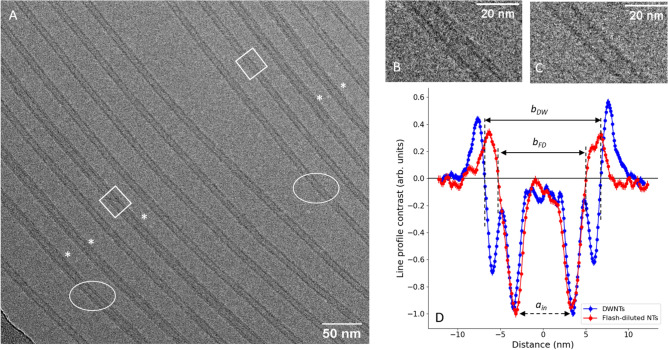
Cryo-TEM image (**A**) containing both DWNTs (marked by *) and flash-diluted NTs, zoomed-in (85X, shown by white oval) sections of DWNTs (**B**), flash-diluted NTs (**C**) and line profiles of DWNTs (blue) and flash-diluted (red) NTs (**D**). The cryo-TEM image (**A**) is recorded at a defocus value of ∼1.5 µm. The line profiles are averaged (Supplementary Information IV) over 30 TEM line profiles, each 22.5 nm in length (two examples are shown by the white rectangles). The line profiles are normalised to a minimum value of −1 for comparison after the background is subtracted (Supplementary Information IV). The solid and dashed black arrows show the two metrics used: the boundary and dip-to-dip distances, respectively, where a_in_ is the inner dip-to-dip distance, while b_dw_ and b_FD_ are boundary distances of DWNTs and flash-diluted NTs, respectively.

To relate the spectroscopic data to the structural data, both DWNTs and flash-diluted NTs were imaged using cryo-TEM. To ensure identical imaging conditions for the purpose of further analysis, we considered the cryo-TEM image containing both DWNTs and flash-diluted NTs (Fig. 3A), and their respective zoomed-in sections are shown in Figs. 3B,C. We emphasise that this was a rare (but otherwise convenient) occasion because the overall efficacy of the flash-dilution process is estimated to be as high as 90%, where the efficacy is calculated as the ratio of the number of flash-diluted NTs to the total number of NTs in all cryo-TEM images (Supplementary Information II). Additional cryo-TEM images obtained in different defocusing conditions are analysed in Supplementary Information III.

**Figure 4:**
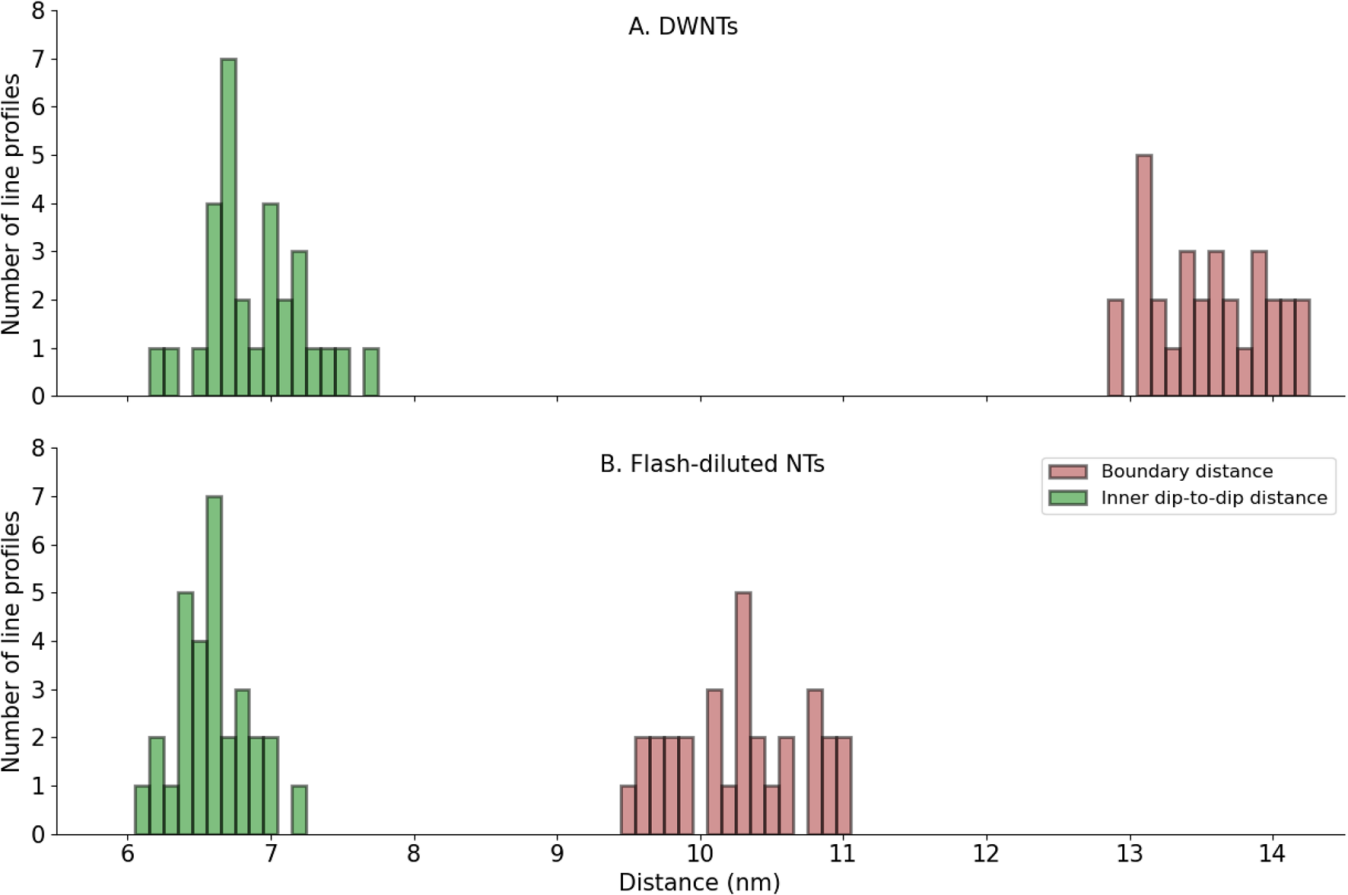
Histograms of boundary distances (brown) and inner dip-to-dip distances (green) of DWNTs (**A**) and flash-diluted NTs (**B**). Thirty line profiles of a total length of ∼0.7 µm were considered for DWNTs and flash-diluted NTs. Each line profile was averaged over a 22.5 nm length and fitted ad hoc with four (DWNT) or two (flash-diluted NT) Gaussians, from which the mean positions were extracted (Supplementary Information IV). The parameters of the histograms are listed in Table 1.

The averaged line profiles of DWNTs and flash-diluted NTs are shown in Fig. 3D, where the minima at ∼ ± 3.5 nm will be referred to as “inner dips”. The boundary distance is traditionally determined from the intersection of the Fresnel fringes with the baseline^13,17,20^, while the dip-to-dip distance is ascertained by finding the inner minima of the line profile^10,13^ (Fig. 3D and Supplementary Information IV). The histograms of boundary and dip-to-dip distances of individual TEM line profiles (i.e. of 22.5 nm segments) are shown in Fig. 4, while Table 1 lists both metrics for DWNTs and flash-diluted NTs. The homogeneity of the DWNTs and flash-diluted NTs can be asserted from the distribution of boundary and dip-to-dip distances of several TEM line profiles (Fig. 4). The standard deviations (SDs) of the histograms of all parameters considered are fairly low—smaller than 5% of the respective values. This indicates that both DWNTs and flash-diluted NTs are fairly homogeneous along the NT length, as well as amongst each other.

**Table 1:** Parameters of the distributions of boundary and dip-to-dip distances of DWNTs and flash-diluted NTs.

	DWNTs		Flash-diluted NTs	
	Mean	SD	Mean	SD
SD Boundary distance b, nm	13.6	0.4	10.2	0.5
Inner dip-to-dip distance a_in_, nm	6.9	0.3	6.6	0.3

The boundary distance of the DWNTs of 13.6 ± 0.4 nm agrees with the values published in the literature^6,13,21,22^. The wall thickness, evaluated from the difference between the boundary distances of DWNTs and flash-diluted NTs, is (13.6–10.2)/2 = 1.7 nm, which agrees well with the size of C8S3 molecule (∼2 nm)^23^ considering the interdigitating aliphatic tails^21^. In other words, the boundary distance of the flash-diluted NTs corresponds to the boundary distance of the DWNTs with the outer layer removed, which strongly suggests that the inner NT remains intact after flash dilution.

### Molecular modelling and TEM image simulation

Different metrics (e.g. boundary distance and dip-to-dip distance) used in the cryo-TEM literature^13,20,21,24^ should be taken with great caution while making connections to the (molecular) macrostructure^21^ because the values they represent might change under different defocusing conditions. In general, peaks and troughs in TEM images cannot be interpreted directly as corresponding to a high and low (projected) charge or mass density^25^, and the literature gives examples of the appearance of ‘ghost’ tubes in multiwalled carbon nanotubes^26^. Therefore, a connection should be made between the molecular structure of the nanotubes and cryo-TEM images. To this end, we used an atomistic molecular model (referred to as the “sample” from here on; for details, see “Materials and methods”) consisting of a single-walled (SW) and a double-walled (DW) section solvated in water. A series of high-resolution TEM (HRTEM) images corresponding to this configuration were generated with the multi-slice algorithm using the program suite abTEM^14^, and line profiles perpendicular to the nanotube axis were obtained (for details, see “Materials and methods”).

**Figure 5:**
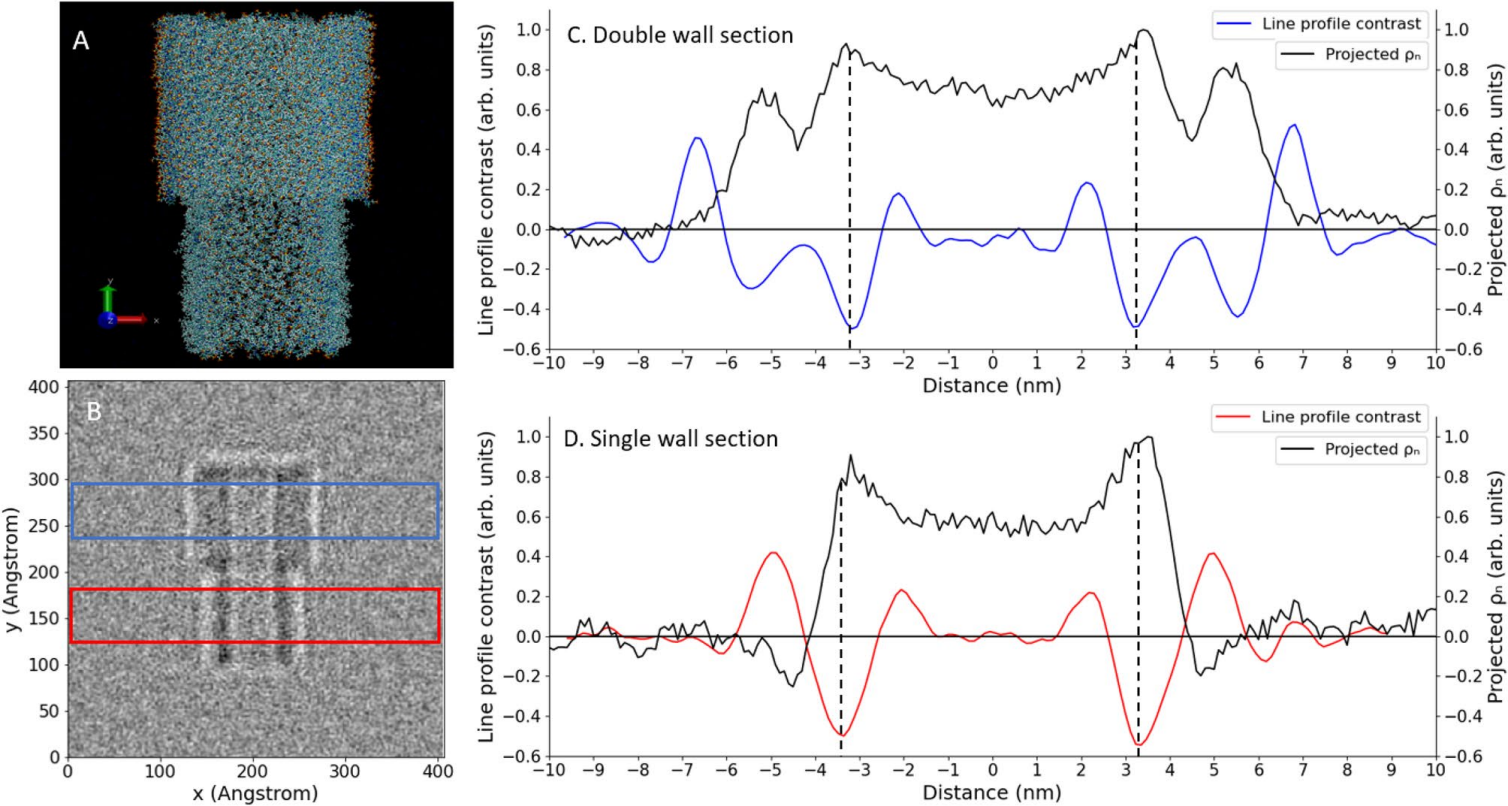
Molecular model of C8S3 NTs (**A**), simulated HRTEM image at defocus of 1.2 µm (**B**) and averaged-contrast line profiles of DW (blue) (**C**) and SW (red) (**D**) sections along with their respective projected nuclear charge densities (ρ_n_). The molecular model (**A**) and simulated HRTEM image (**B**) show both DW and SW sections, which are highlighted in the latter by blue and red rectangles, respectively. Five line profiles of 20 nm each from the simulated HRTEM image are averaged and smoothened by taking a rolling average over seven points (**C**,**D**). The line profiles (projected nuclear densities) are normalised to a minimum (maximum) value of −0.5 (+ 1) for the ease of comparison. In panels (**C**) and (**D**), the dashed lines show the positions of the inner dips (∼ ± 3.5 nm) of the line profiles, which are reasonably close (within 0.5 nm) to the positions of the inner maxima of the projected nuclear densities.

Figures 5A,B show C8S3 molecules arranged in a tubular structure for the molecular model and the simulated HRTEM image of the sample, respectively. Simulated HRTEM line profile contrasts for DW and SW sections, as well as the corresponding profiles of the projected nuclear charge density (for details, see Supplementary Information VII) of the atomistic model (including all solvent and counter ions), are depicted in Fig. 5C,D, respectively. The projected nuclear charge densities of the DW and SW sections overlap closely, with the peak in the density at almost the same distance from the centre of the tube (∼3.3 nm). Furthermore, the positions of the inner maxima of the projected nuclear charge densities (shown by dashed lines) are reasonably close (within 0.5 nm) to the positions of the inner dips (i.e. the minima of the line profiles at ∼ ± 3.5 nm) for both the DW and SW sections (Fig. 5C,D).

The contrast line profiles of DWNTs and flash-diluted NTs obtained from the experimental cryo-TEM images (Fig. 3D) bear a close resemblance to the simulated line profile contrasts (Fig. 5C,D). The overall shapes of the line profiles are well- reproduced, and the inner dips nicely coincide in the experiment (for DWNTs and flash-diluted NTs, Fig. 4, green histograms) and theory (for DW and SW samples, Fig. 5C,D). In noting that the SW section was obtained by simply removing the molecules of the outer NT, without changing the molecular structure of the inner NT, we conclude that the flash-dilution does not affect the inner wall. The positions of the dips slightly drift for different values of the defocus (Supplementary Information III, Fig. S8); nevertheless, they do so in much the same fashion for the SW and inner DW sections (Supplementary Information III, Fig. S9). The deviations in the particular defocus values (1.2 µm for theoretical calculations vs. ∼1.5 µm in experiment) and distance scaling (∼10% between the experiment and calculations, not shown explicitly) may be due to differences in samples thickness, finite transversal coherence of the electron beam, and accuracy of the potential used in the multislice method. Nevertheless, the general trends seen in the simulated HRTEM images support our conclusion that the inner wall survives flash-dilution intact.

### Blue shift in the flash-diluted NT spectrum

Finally, we comment on a small, but noticeable, blue shift of ∼40 cm^−1^ observed in the absorption spectrum of the inner NT after flash-dilution (Fig. 2). This blue shift was earlier hypothesised to be attributed to either the change in the inner NT diameter after flash-dilution^18^ or shortening of the length of NTs (to 100 nm or shorter) due to the nano-confinement of excitons^27^. As shown herein, the flash-dilution process does not change the inner NT. Furthermore, when considering the sight of view of the cryo-TEM grid, no significant changes were observed in the lengths of flash-diluted NTs (Supplementary Information V, Fig. S12). The latter finding corroborates an earlier conclusion based on the high degree of geometrical alignment of the flash-diluted NTs in the microfluidics channel^18^. Accordingly, the blue spectral shift is most likely because the inner NT begins to be directly exposed to the solvent, which causes a change in the dielectric constant of the surrounding. This is in line with the previous finding^5^ on the effect of the solvent on the DWNT system, where the absorption spectrum of DWNTs prepared via the alcoholic route was blue-shifted compared to DWNTs prepared via the direct route (i.e. no methanol in the solvent).

## Conclusions

By using microfluidics, optical spectroscopy and cryo-TEM, we have been able to image the transient inner NTs before the outer NTs reform under strong non-equilibrium conditions. Experimental cryo-TEM images were compared with simulated HRTEM images obtained from molecular modelling of the C8S3 NT system. The close correspondence between the two allowed us to conclude that the inner NT remains unchanged after flash-dilution. The high homogeneity of the inner NT was also shown to be preserved through the flash-dilution process, which proves that flash-dilution selectively dissolves only the outer NT, leaving the molecular structure of the inner NT mostly intact. This conclusion opens up a new research pathway to introduce additional nanostructure(s) (such as e.g. quantum dots) to the inner NT before the outer NT begins to reform, to alter the electronic coupling between the walls or the exciton diffusion length.

## Materials and methods

### Sample preparation

C8S3 dye (3,3’-bis(2-sulfopropyl)-5,5’,6,6’-tetrachloro-1,1’-dioctylbenzimidacarbocyanine, MW = 903 g/mol) was purchased from FEW Chemicals (Wolfen, Germany), and was used as received. C8S3 DWNTs were prepared via the alcoholic route detailed in Ref.^5^. In brief, 2.32 mM monomer stock was prepared by dissolving C8S3 dye molecules in methanol (Biosolve BV). This was followed by the addition of milli-Q water to the monomer stock solution in the ratio 1:0.26 v/v. There was an immediate change in colour from orange to pink, which is an indication of self-assembly of the monomers, resulting in the formation of DWNTs. The resulting solution was stored for 15 – 18 h at room temperature in a dark place. Following this, 1 mL milli-Q water was added, resulting in a final C8S3 dye concentration of 0.267 mM, and 9% (w/w) methanol in DWNT solution.

### Microfluidic flash-dilution

Flash-dilution was carried out by mixing the DWNT solution with a mixture of methanol and water (1:1 v/v) in a borosilicate microfluidic teardrop mixer (Micronit), the geometry of which ensures controlled and efficient mixing at low Reynolds numbers (RE < 100) with a clearly defined beginning of the flash-dilution process. Two syringe pumps (New Era, model NE–300) were used to pump the DWNT solution and the methanol–water mixture into the microfluidic mixer at a flowrate ratio of 1:1.6, respectively, with a total flowrate of 2.6 mL/h. After mixing, the flash-diluted NTs flowed to the microfluidic flow cell (Micronit), where the absorption spectrum was constantly monitored in a compact, portable, home-built absorption setup, which can be operated in the vicinity of the cryo-TEM freezing station. A white LED light as a light source and an Ocean Optics USB-400 spectrometer were used. The total time for the reagents to flow from the mixer to the end of the flow cell was ∼10 s. The flash-diluted NTs were collected at the end of the flow cell and were frozen rapidly within 30 s of collection.

### Cryogenic transmission electron microscopy

Flash-diluted NTs were frozen (following the protocol described in Ref.^13^) ∼40 s after the beginning of the flash-dilution process. Firstly, around 3 µL of flash-diluted NT was deposited on a hydrophilised copper grid with a holey carbon film (quantifoil 3.5/1). Then, a thin layer of the sample (∼100 nm) was formed by blotting off the excess for 5 s. This was followed by rapidly vitrifying the grid at −184 °C in liquid ethane using a Vitrobot (FEI Eindhoven). For imaging, a FEI Tecnai T20 transmission electron microscope with a LaB6 cathode operating at 200 keV was used, where the vitrified grids were placed in a cryotransfer holder (Gatan model 626). The cryo-TEM images were recorded using an UltraScan 4000 UHS CDD camera (Gatan, Pleasanton) in the low-dose mode. Four samples each of DWNTs and flash-diluted NTs were imaged at different defocus and magnification. From these, two micrographs of DWNTs and three micrographs of flash-diluted NTs were arbitrarily used in the main text and Supplementary Information. With the magnification value of 100,000 × used in the reported images (unless stated otherwise), the spatial resolution is estimated as ∼0.5 nm, while the uncertainty in the defocus value of the microscope is ∼0.5 µm. Additional imaging parameters are listed in Table 2.

**Table 2:** Cryo-TEM imaging parameters.

Pixel size	2.24 Å
Exposure time	1 s
Dose rate	50 e/Å/s
Total dose	50 e/Å
Defocus	−1500 nm and −3000 nm

### Data processing

The cryo-TEM images were analysed with Fiji Image J2 software; the details are discussed in Supplementary Information IV.

### Molecular dynamics model

The C8S3 nanotube model from Bondarenko et al.^28^ was used as a starting structure for our simulations. The specific model reproduced the experimental absorption spectra of the C8S3 nanotubes and maintained its tubular structure during a production phase of 100 ns. The process of constructing C8S3 nanotubes was based on creating 2D lattices from different unit cells and rolling them into cylinders with specific radii and rolling angles (for details, see Supplementary Information VI). The radius for the inner wall cylinder of this model was 3.72 nm and the rolling angle was 30.96°, whereas, for the outer wall cylinder, these values were 5.49 nm and 31.53°, respectively. The details are described in Refs.^21,28^.

To calculate the line profile contrasts, a nanotube piece of 20 nm length was cropped from the C8S3 nanotube model (originally 75 nm). Then, the C8S3 molecules of the lower half of the nanotube were removed. The resulting structure constituted a complete C8S3 nanotube of 10 nm attached to an exposed C8S3 inner tube of 10 nm (Fig. 5A). The cropped nanotube was solvated in the centre of a 40 × 40 × 40 nm box and Na^+^ ions were added to neutralise the charge of the system. To make sure the dimensions of the inner wall of the tube were the same in the SW and DW sections, position restraints were applied to the chromophore of the inner-wall C8S3 molecules. The total number of C8S3 molecules was 1068 (inner NT: 604 & outer NT: 464), and the total number of atoms was 6,807,144. Further details can be found in Supplementary Information VI and in Refs.^21,28^.

### TEM image simulation

The atomic positions of all atoms in the model (∼6.8 million in total) were used to generate simulated high-resolution TEM (HRTEM) images using the modules of the abTEM package^14^, by numerically propagating a plane wave with a real-space resolution of 1.0 Angstrom across the electrostatic potential using the multislice algorithm^29^. The Lobato parameterisation^30^ of the atomic potentials was used, with a slice thickness of 0.1 Angstrom and with exact integration in the direction of the plane wave over the slice. The exit wave was subsequently converted to an HRTEM image by applying a contrast transfer function (CTF). The CTF settings were close to those of the electron microscope used in the experiment (see Supplementary Information VII), but we varied the defocus. Line profile contrasts for the SW and DW sections were generated from the image by averaging five line profiles each of 20 nm in length across the tube in the SW and DW sections. The method used to obtain nuclear charge density profiles is elaborated in Supplementary Information VII.

## Supplementary Information

Supplementary Information.

## Data Availability

All data generated or processed during this study are available from the corresponding author upon reasonable request.

## References

[1] Mirkovic T (2017). Light absorption and energy transfer in the antenna complexes of photosynthetic organisms. Chem. Rev..

[2] Chenu A, Scholes GD (2015). Coherence in energy transfer and photosynthesis. Annu. Rev. Phys. Chem..

[3] Eisele DM, Knoester J, Kirstein S, Rabe JP, Vanden Bout DA (2009). Uniform exciton fluorescence from individual molecular nanotubes immobilised on solid substrates. Nat. Nanotechnol..

[4] Haedler AT (2015). Long-range energy transport in single supramolecular nanofibres at room temperature. Nature.

[5] Von Berlepsch H, Kirstein S, Hania R, Pugžlys A, Böttcher C (2007). Modification of the nanoscale structure of the J-aggregate of a sulfonate-substituted amphiphilic carbocyanine dye through incorporation of surface-active additives. J. Phys. Chem. B.

[6] Eisele DM (2014). Robust excitons inhabit soft supramolecular nanotubes. Proc. Natl. Acad. Sci. United States Am..

[7] Günther LM (2016). Structure of light-harvesting aggregates in individual chlorosomes. J. Phys. Chem. B.

[8] Scholes GD, Fleming GR, Olaya-Castro A, Van Grondelle R (2011). Lessons from nature about solar light harvesting. Nat. Chem..

[9] Brixner T (2005). Two-dimensional spectroscopy of electronic couplings in photosynthesis. Nature.

[10] Eisele DM (2012). Utilising redox-chemistry to elucidate the nature of exciton transitions in supramolecular dye nanotubes. Nat. Chem..

[11] Kriete B (2019). Interplay between structural hierarchy and exciton diffusion in artificial light harvesting. Nat. Commun..

[12] Clark KA, Krueger EL, Vanden Bout DA (2014). Direct measurement of energy migration in supramolecular carbocyanine dye nanotubes. J. Phys. Chem. Lett..

[13] Kriete B (2017). Steering self-assembly of amphiphilic molecular nanostructures via halogen exchange. J. Phys. Chem. Lett..

[14] Madsen J, Susi T (2020). abTEM: ab Initio Transmission Electron Microscopy Image Simulation. Microsc. Microanal..

[15] Berlepsch HV, Ludwig K, Kirstein S, Böttcher C (2011). Mixtures of achiral amphiphilic cyanine dyes form helical tubular J-aggregates. Chem. Phys..

[16] Clark KA, Cone CW, Vanden Bout DA (2013). Quantifying the polarisation of exciton transitions in double-walled nanotubular J-aggregates. J. Phys. Chem. C.

[17] Didraga C (2004). Structure, spectroscopy, and microscopic model of tubular carbocyanine dye aggregates. J. Phys. Chem. B.

[18] Kriete B, Feenstra CJ, Pshenichnikov MS (2020). Microfluidic out-of-equilibrium control of molecular nanotubes. Phys. Chem. Chem. Phys..

[19] Clark KA, Krueger EL, Vanden Bout DA (2014). Temperature-dependent exciton properties of two cylindrical J-aggregates. J. Phys. Chem. C.

[20] Qiao Y (2014). In situ synthesis of semiconductor nanocrystals at the surface of tubular J-aggregates. J. Mater. Chem. C.

[21] Patmanidis I (2020). Structural characterisation of supramolecular hollow nanotubes with atomistic simulations and SAXS. Phys. Chem. Chem. Phys..

[22] Von Berlepsch H, Kirstein S, Böttcher C (2002). Effect of alcohols on J-aggregation of a carbocyanine dye. Langmuir.

[23] Prokhorov V, Petrova M, Kovaleva N, Demikhov E (2014). Atomic force and scanning near-field optical microscopy study of carbocyanine dye J-aggregates. Curr. Nanosci..

[24] Eisele DM (2010). Photoinitiated growth of sub-7 nm silver nanowires within a chemically active organic nanotubular template. J. Am. Chem. Soc..

[25] Fultz B, Howe J (2013). High-Resolution TEM Imaging, 521–586.

[26] Hayashi T (2006). TEM image simulation study of small carbon nanotubes and carbon nanowire. Carbon.

[27] Bondarenko AS, Jansen TL, Knoester J (2020). Exciton localisation in tubular molecular aggregates: Size effects and optical response. J. Chem. Phys..

[28] Bondarenko AS (2020). Multiscale modeling of molecular structure and optical properties of complex supramolecular aggregates. Chem. Sci..

[29] Cowley JM, Moodie AF (1957). The scattering of electrons by atoms and crystals: I: A new theoretical approach. Acta Cryst..

[30] Lobato I, Van Dyck D (2014). An accurate parameterisation for scattering factors, electron densities and electrostatic potentials for neutral atoms that obey all physical constraints. Acta Crystallogr. Sect. A: Foundations Adv..

