# Supplementary material for: Cryogenic TEM imaging of artificial light harvesting complexes outside equilibrium
Source: Sci Rep. 2022 Apr 1;12:5552. doi: 10.1038/s41598-022-09496-z (PMC8975939; doi:10.1038/s41598-022-09496-z)
Supplement: Supplementary file 1 — Supplementary Information. [file 41598_2022_9496_MOESM1_ESM.docx]

**Supplementary information**

**Cryogenic TEM Imaging of Artificial Light Harvesting Complexes Outside Equilibrium**

Sundar Raj Krishnaswamy^1^, Ivo A. Gabrovski^1^, Ilias Patmanidis^2^, Marc C.A. Stuart^2^, Alex H. de Vries^2^, and Maxim S. Pshenichnikov^1,^*

^1^ Zernike Institute for Advanced Materials, University of Groningen, Nijenborgh 4, 9747 AG Groningen, the Netherlands

^2^ Groningen Biomolecular Sciences and Biotechnology Institute, Nijenborgh 7 9747 AG Groningen, the Netherlands

* corresponding author. Email : m.s.pchenitchnikov@rug.nl

**Supplementary information**

Section I. Presence of bundles …………………………………………………………………………………………….. 2

Section II. Efficiency of flash-dilution process ………………………………………………………………………. 3

Section III. Experimental and simulated TEM line profiles at different defocus conditions ….. 4 - 8

Section IV. Data processing of cryo-TEM images ………………………………………………………………….. 9 - 10

Section V. Length of NTs after flash-dilution ………………………………………………………………………… 11

Section VI. Molecular model building and MD simulation ……………………………………………………. 12

Section VII. Projected nuclear charge density and TEM image calculation with abTEM ………… 13 – 14

I. Presence of bundles

Fig. S1 shows the spectra of DWNTs, and flash diluted NTs, where the spectrum of the latter is red shifted by ~40 cm^-1^ to overlap with the peak at ~600 nm. In the spectrum of DWNTs, the shoulder at ~604 nm (~16550 cm^-1^) is due to the contribution of bundles^1,2^ (i.e, aggregation of several NTs with an outer layer of self-assembled dye molecules^3^).


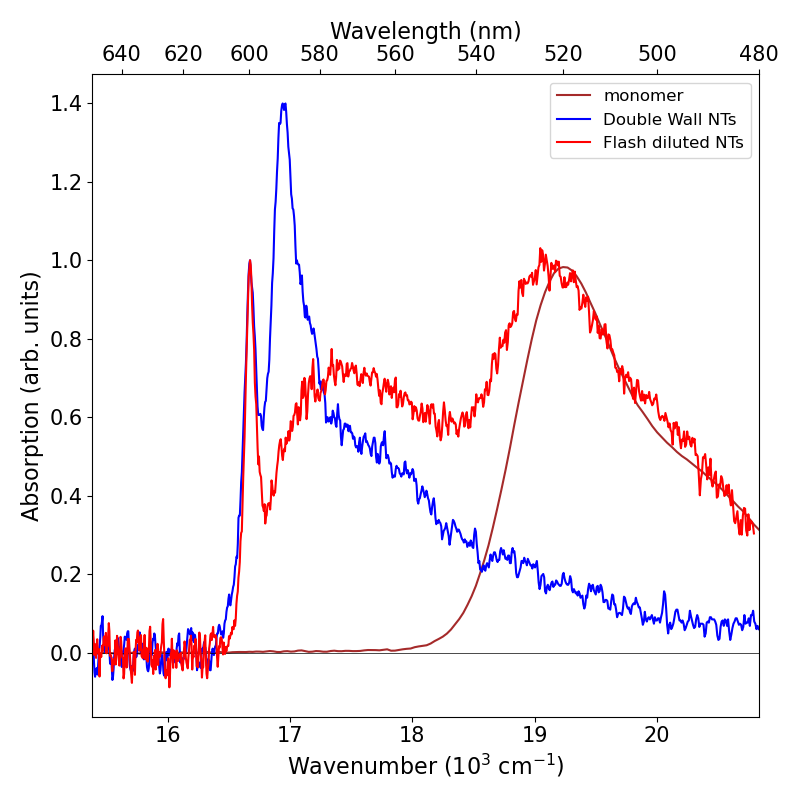


**Figure S1**. Absorption spectra of DWNTs (red), and flash-diluted NTs (red). Unlike Fig. 2 in the main text, the spectrum of flash-diluted NTs is red-shifted ~40 cm for the peaks at ~600 nm to overlap. The widths of the peak ~600 nm are similar, while a small shoulder at ~604 nm is also observed in the spectrum of DWNTs which is due to the formation of bundles.

Fig. S2 shows bundles which were observed in the cryo-TEM image of DWNTs, where the sample of DWNTs was prepared following the same procedure as described in the main text (see Materials and Methods). In ~ 35 unique DWNTs images, 1 bundle was observed.


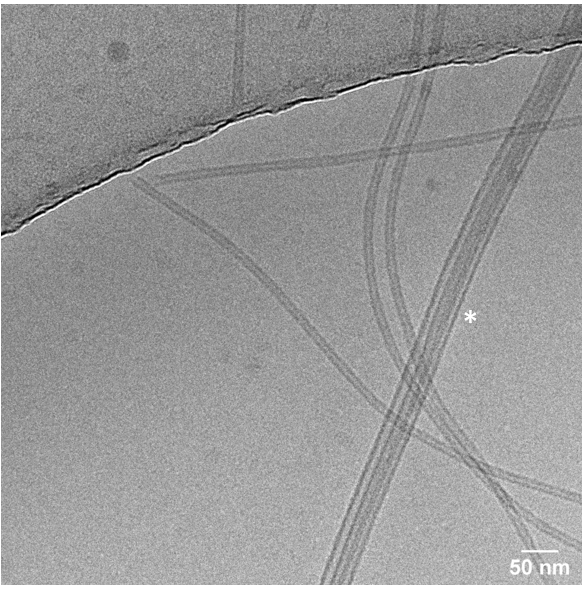


**Figure S2.** Cryo-TEM image of DWNTs where bundles (*) are also observed.

II. Efficacy of flash-dilution process

We begin with estimation of the amount of DWNTs which are completely dissolved after flash dilution. The relative change in the amplitude of the peak at ~600 nm^4^ which corresponds to the inner tube absorption, is by a factor of ~4.5 (Fig. S3). DWNTs and 1 : 1 (v/v) methanol-water mixture are mixed in the ratio 1:1.6, respectively (see Materials and Methods section in the main text) which results in an additional “dilution” factor of 2.6. Therefore, ~40% of DWNTs are completely dissolved by the flash-dilution process so that ~60% of the parent DWNTs survive flash dilution.


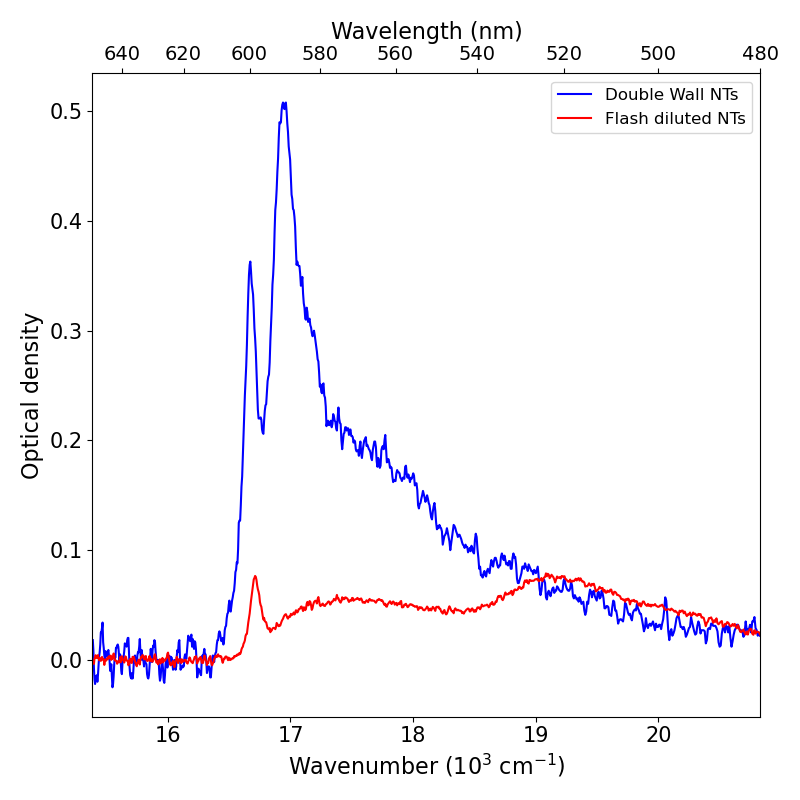


**Figure S3.** Absorption spectra of DWNTs (blue), and flash-diluted NTs (red). The amplitudes of the peak at ~600 nm is 0.36 (DWNTs), and 0.08 (flash-diluted NTs); the relative change in the amplitude of the peak after flash-dilution is ~40% taking into account the effect of dilution (dilution factor is 2.6).

Furthermore, considering NTs which were not completely dissolved, the efficacy of flash-dilution is estimated as the ratio of number of flash-diluted NTs observed to the total number of NTs in all the cryo-TEM images. In total, ~ 49 unique NTs were imaged using cryo-TEM out of which ~ 43 NTs were flash-diluted, while the rest ~ 6 NTs were double-walled (mostly situated in a single image, Fig. 3A in the main text). Thus, we estimate the efficiency of the flash-dilution process performed as ~90%.

III. Experimental and simulated TEM line profiles at different defocus conditions

Experimental line profiles:

Fig. S4 A shows DWNTs and flash-diluted NTs in the same cryo-TEM image as in Fig. 3A in the main text, but at different defocus setting of the microscope (3 µm). Fig. S4 B and S4 C show the zoomed-in sections of DWNTs and flash-diluted NTs, respectively, while Fig. S4 D shows the corresponding normalised averaged line profiles. Unlike the zoomed-in sections in Figs. 3B and 3C (main text), the edges of the NTs in Figs. S4 B and S4 C are not sharp at all. This effect is highlighted in Fig. S4 D (also see Fig. S6), where the outer dips (~ ± 6nm) of DWNTs are not clearly resolved unlike the line profiles shown in Fig. 3D (main text), which is the influence of defocus setting of the microscope. Nonetheless, the inner dips (~ ± 3 nm) of both DWNTS and flash-diluted NTs coincide, which agrees well with the simulated line profiles from HRTEM images (*vide infra*, Fig. S9) and thus, strengthens the conclusion that the size of the inner NTs are unchanged after the flash-dilution process.


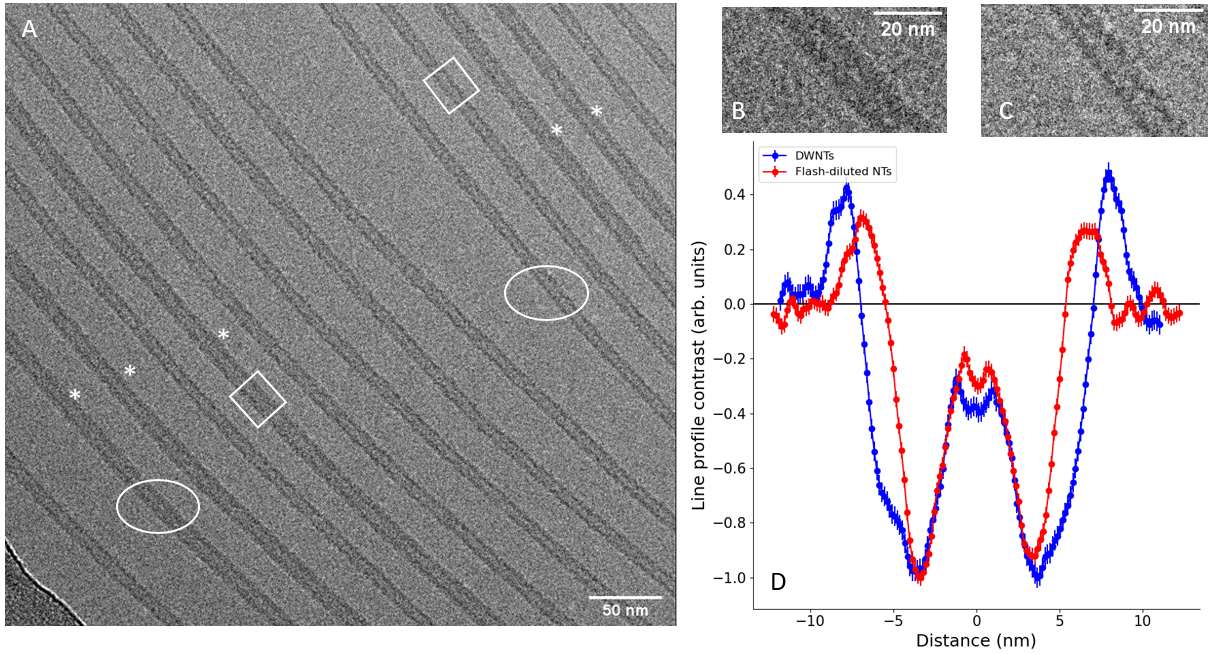


**Figure S4.** Cryo-TEM image of both DWNTs and flash-diluted nanotubes (A), their respective zoomed in sections (B and C), and averaged line profiles of DWNTs and flash-diluted NTs (D). The image (A) is the same image as Fig. 3A but is recorded at a different defocus (~3 µm). Similarly to Fig. 3 in the main text, 30 TEM line profiles of 22.5 nm are considered from both DWNTs (marked by *) and flash diluted NTs and are averaged after linear-interpolating onto the same x-scale. The line profiles are normalised for the ease of comparison after subtracting the background (Supplementary Information IV).


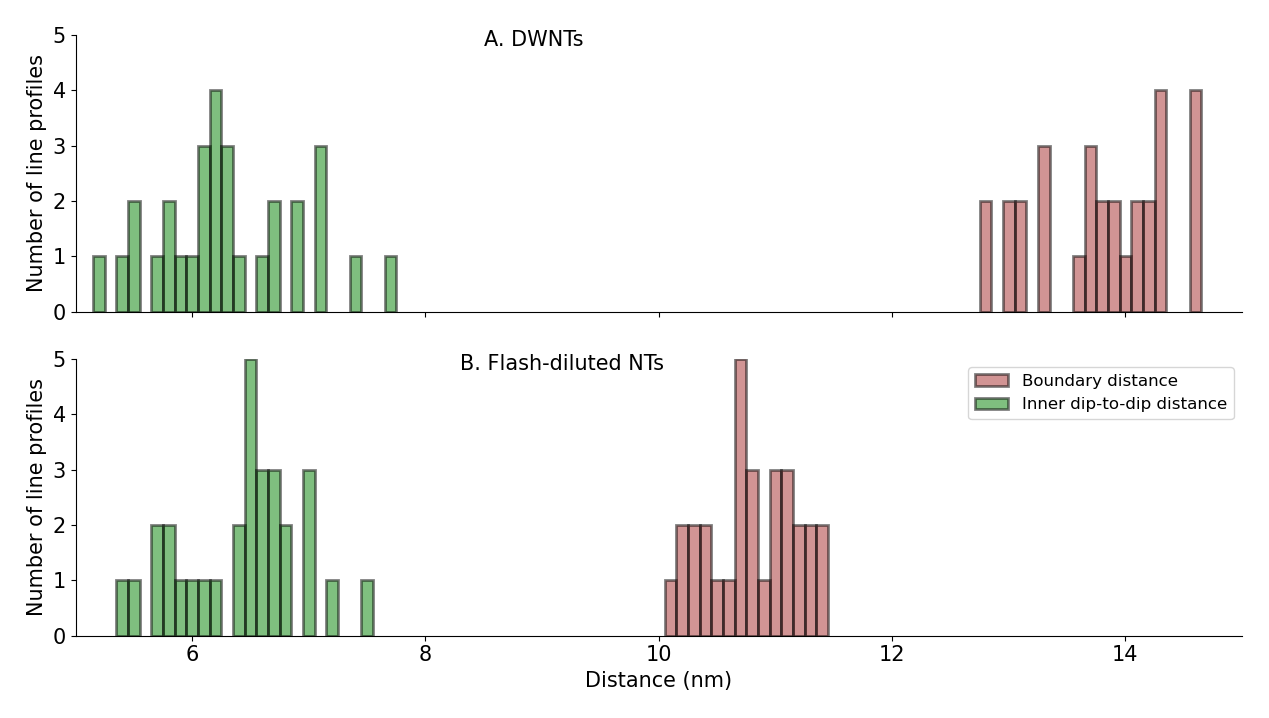


**Figure S5.** Distribution of boundary (brown) and inner dip-to-dip (green) distances of DWNTs (A) and flash-diluted NTs (B). Similar to Fig. 4 in the main text, 30 line profiles each 22.5 nm in length are considered from DWNTs and flash-diluted NTs. The line profiles are modelled following the same procedure as done in Fig. 4 (for details, see Supplementary Information IV) to obtain the boundary and inner dip-to-dip distances. The parameters of the distribution are shown in Table S1.

**Table S1.** Parameters of the distributions of boundary and dip-to-dip distances of DWNTs and flash-diluted NTs shown in Fig. S4.

|  | **DWNTs** | | **Flash-diluted NTs** | |
| --- | --- | --- | --- | --- |
|  | Mean | SD | Mean | SD |
| Boundary distance $b$, nm | 13.8 | 0.6 | 10.8 | 0.4 |
| Inner dip-to-dip distance$a_{in}$, nm | 6.3 | 0.6 | 6.3 | 0.6 |

The distribution of boundary and inner dip-to-dip distances is shown in Fig. S5 and the parameters of the distribution is shown in Table S1. The values of boundary distance and inner dip-to-dip distance are in good agreement with the values in Table 1 in the main text, while the SD of both histograms are slightly higher. This is due to lower resolution and hence poorer visibility of the edges which causes systematic errors in modelling of profiles to calculate dip-to-dip and boundary distances (see Supplementary Information IV). However, SD values of the histograms of both distances are fairly low (< 7%), which further agrees with the fact that the DWNTs and flash-diluted NTs are fairly homogeneous along the NT length as well as amongst each other.

Next, the line profiles of DWNTs and flash-diluted NTs shown in Fig. 3D (defocus of 1.5 µm) and Fig. S4 D (defocus of 3 µm) are compared in Fig. S6. The line profiles of DWNTs (Fig. S6 A) clearly show two outer (~ ± 6 nm), and two inner (~ ± 3 nm) dips at defocus of 1.5 µm (shown by triangles), while the two outer dips are not well resolved at defocus of 3 µm (shown by circles). This effect of defocus values on modulation of dips also agrees with simulated line profiles generated from (simulated) HRTEM images (see Fig. S8). Furthermore, the two inner dips are well resolved in the line profiles of flash-diluted NTs (Fig. S6 B) at both the defocus values. Therefore, relating a given line profile to DWNTs or flash-diluted NTs solely based on the number of dips should be done with care and thus, there is a need to develop a relation between the molecular structure of NTs, the corresponding TEM images and line profiles, as done in Fig. 5 (main text), and Fig. S8.


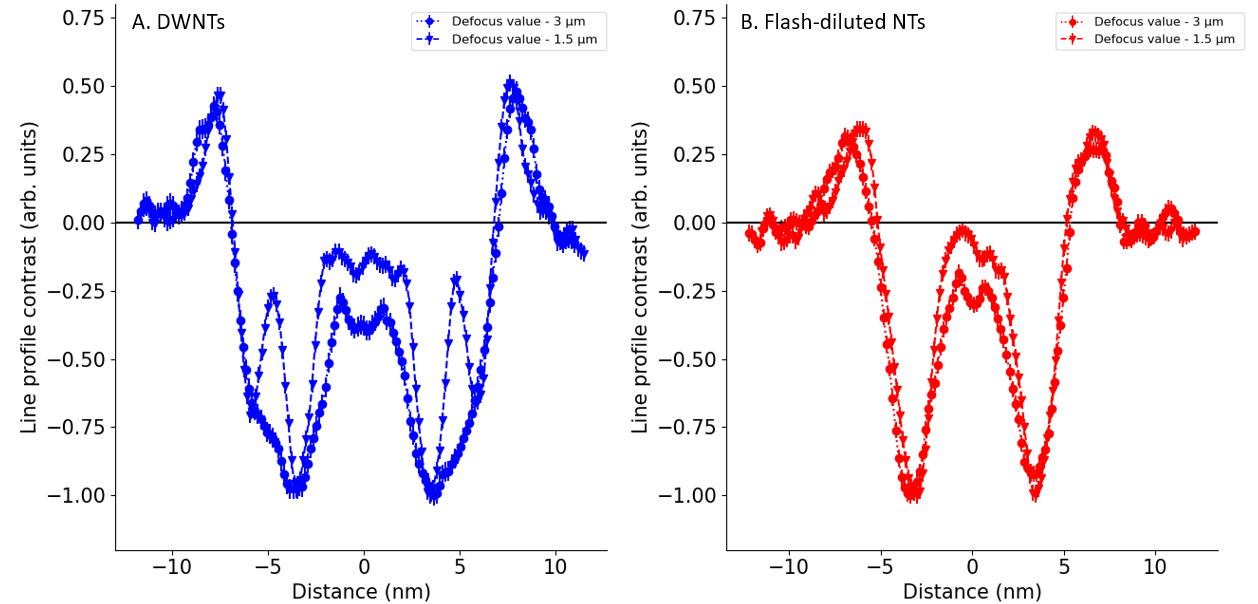


**Figure S6.** Comparison of line profiles of DWNTs (A) and flash-diluted NTs (B) at defocus values of 3 µm (circles ) and 1.5 µm (triangles). The line profiles are normalised to a minimum value of -1 for the ease of comparison. The line profiles and cryo-TEM images at the former defocus value is discussed in the main text (Fig. 3), while the latter is discussed is Fig. S5.

Simulated line profiles

Similar to Fig. 5B in the main text, HRTEM images of double wall (DW) and single wall (SW) sections at different defocus setting were generated from atomistic molecular model using the program suite abTEM (for details, refer to Supplementary Information VII), and are shown in Fig. S7, where DW and SW sections are highlighted by blue and red rectangles, respectively.


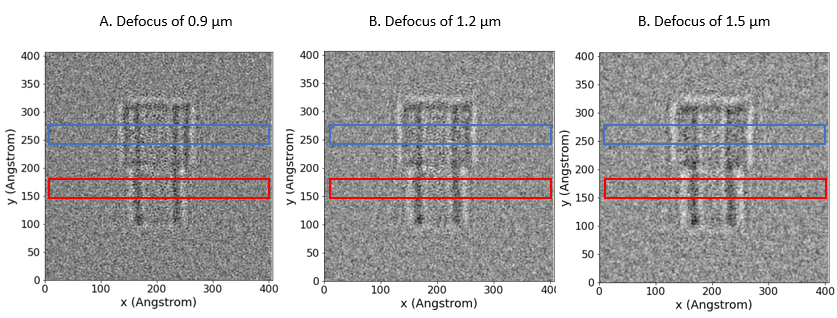


**Figure S7.** Simulated HRTEM images of at defocus values of 0.9 µm (A), 1.2 µm (B), and 1.5 µm (C), where DW and SW sections are shown by blue and red rectangles, respectively.

The line profiles are obtained from the simulated HRTEM image as detailed in Fig. 5 in the main text. The averaged contrast line profiles of DW and SW sections at different defocus values are shown in Fig. S8 along their respective nuclear density distributions. Here, it can be noted that the modulation of dips is strongly influenced by the defocus setting of the microscope, which is also observed experimentally, up to the point that the modulation in the DW line profile is smeared off (Fig. S4D, Fig. S6 A, and Fig. 3D in the main text). Next, the position of the inner dips of both DW and SW sections fairly overlap (± 0.3 nm) at different defocus settings, while the crossing points of the line profiles with the baseline (shown by black circles, Fig. S8 also roughly coincide (within 0.3 nm). Furthermore, although the inner dip-to-dip distances change by ~0.3 nm with defocus settings for both DW and SW sections (Fig. S9), they do so in a similar fashion which allows their direct comparison provided that images of DWNT and flash-diluted NTs are obtained at identical defocussing values (as is the case in Fig. 3 in the main text). This further reinforces the claim that the size of the inner wall is unaffected by the flash-dilution process.


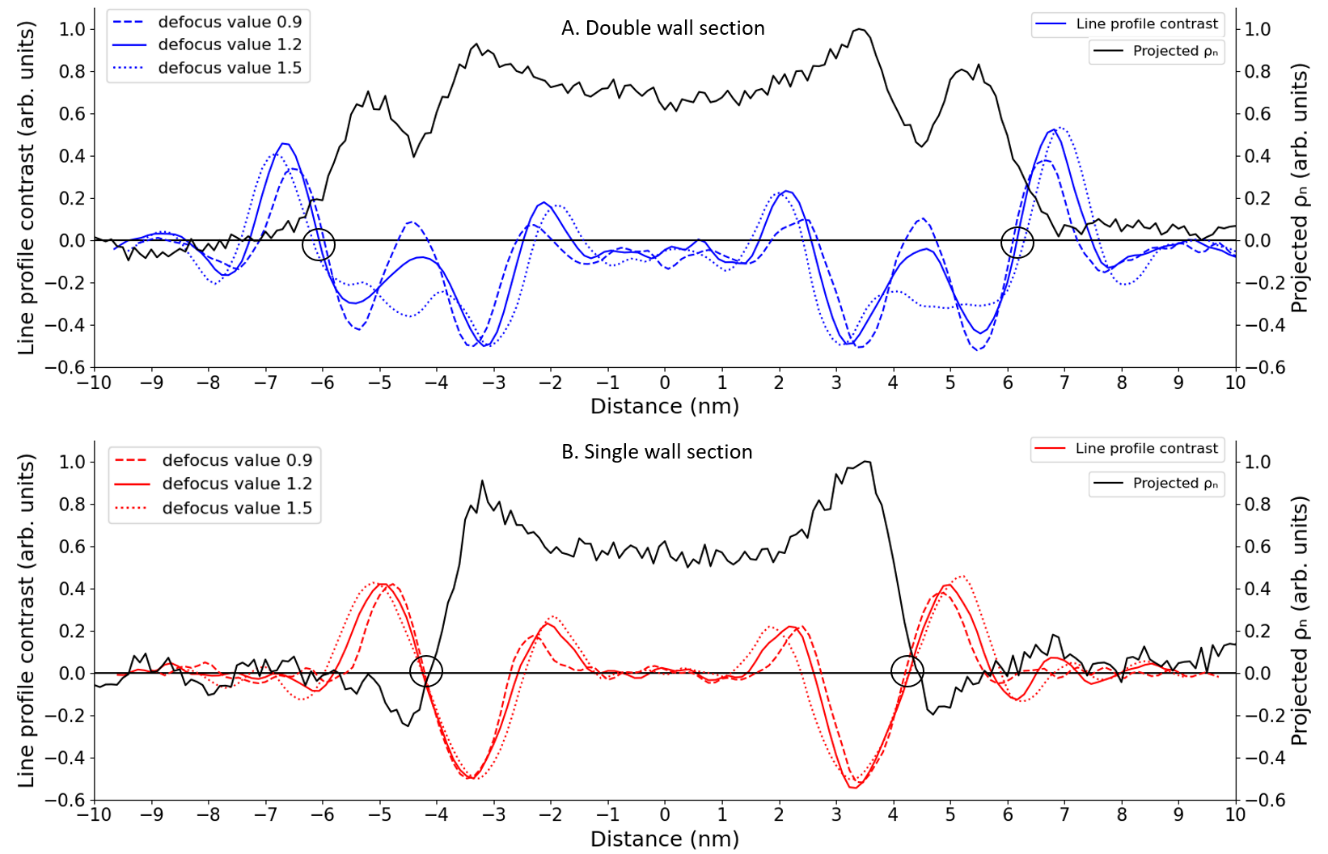


**Figure S8.** Averaged line profile contrast of DW (A) and SW sections (B) at different defocus setting shown along with their respective nuclear density distribution. The profiles are plotted in the same way as detailed in the main text (Fig. 5C). The line profile contrast (projected nuclear density) is normalised to a minimum value of -0.5(+1) for the ease of comparison. The black circles highlight crossing points of the line profiles with the baseline.


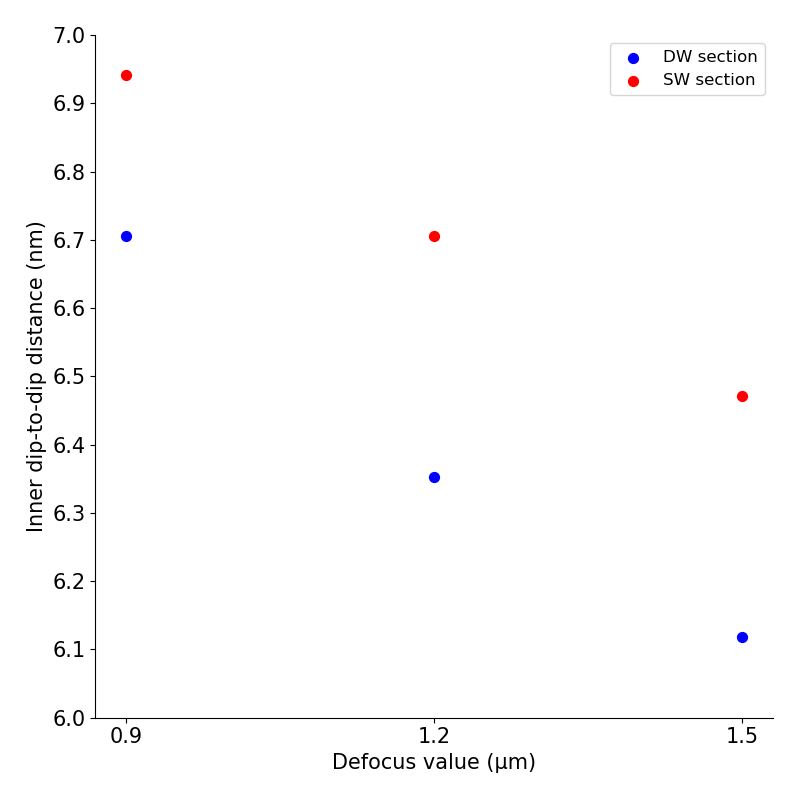


**Figure S9.** Inner dip-to-dip distance of DW (blue dots) and SW (red dots) sections at different defocus setting of the microscope. Although the distance of DW and SW sections slightly vary (by ~0.3 nm) at different defocus setting, they do so in a synchronous fashion.

IV. Data processing of cryo-TEM images

The cryo-TEM images were analysed using the Fiji Image J2 software. The initial line profiles were obtained from cryo-TEM images as detailed in Fig. 3D in the main text. Individual line profiles were obtained by averaging over a length of 22.5 nm along the NT, which on one hand presents a good compromise between the signal and noise, and on the other hand allows avoiding the NT bends.

Figs. S10 and S11 detail the analysis of the individual line profiles of DWNTs and flash diluted NTs, respectively. First, the baseline approximated with a linear function (Fig. S10 A, Fig. S11 A) was subtracted (Fig. S10 B, Fig. S11 B). Next, the line profiles of DWNTs and flash diluted NTs were ad hoc fitted with four (Fig. S11 C) and two Gaussian functions (Fig. S11 C), respectively. Following this, the intersection of the profiles with the baseline is determined by linear interpolation, and profiles were centered around the them (Fig. S10 D, Fig. S11 D). Then, the line profiles of DWNTs and flash-diluted NTs were linearly interpolated between -12 nm and +12 nm and were averaged.


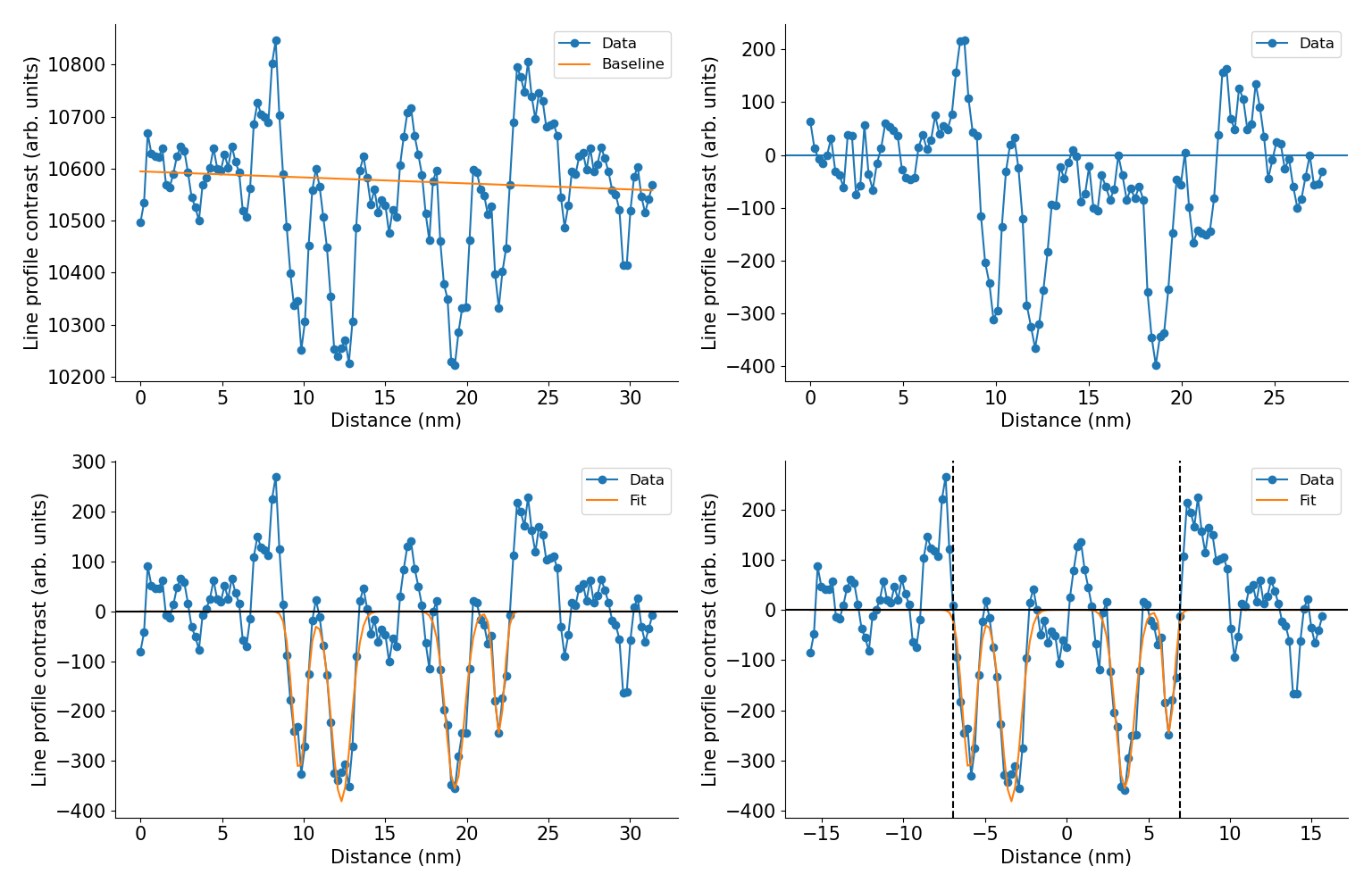


**Figure S10.** Data processing of a representative line profile of DWNTs. A. Modelling the baseline using a linear function. B. Subtracting the baseline from the line profile. C. Fitting the line profile with four Gaussian functions. D. Finding the intersection of the line profile with the baseline by interpolation (shown by dashed lines), and centering the line profiles between them.


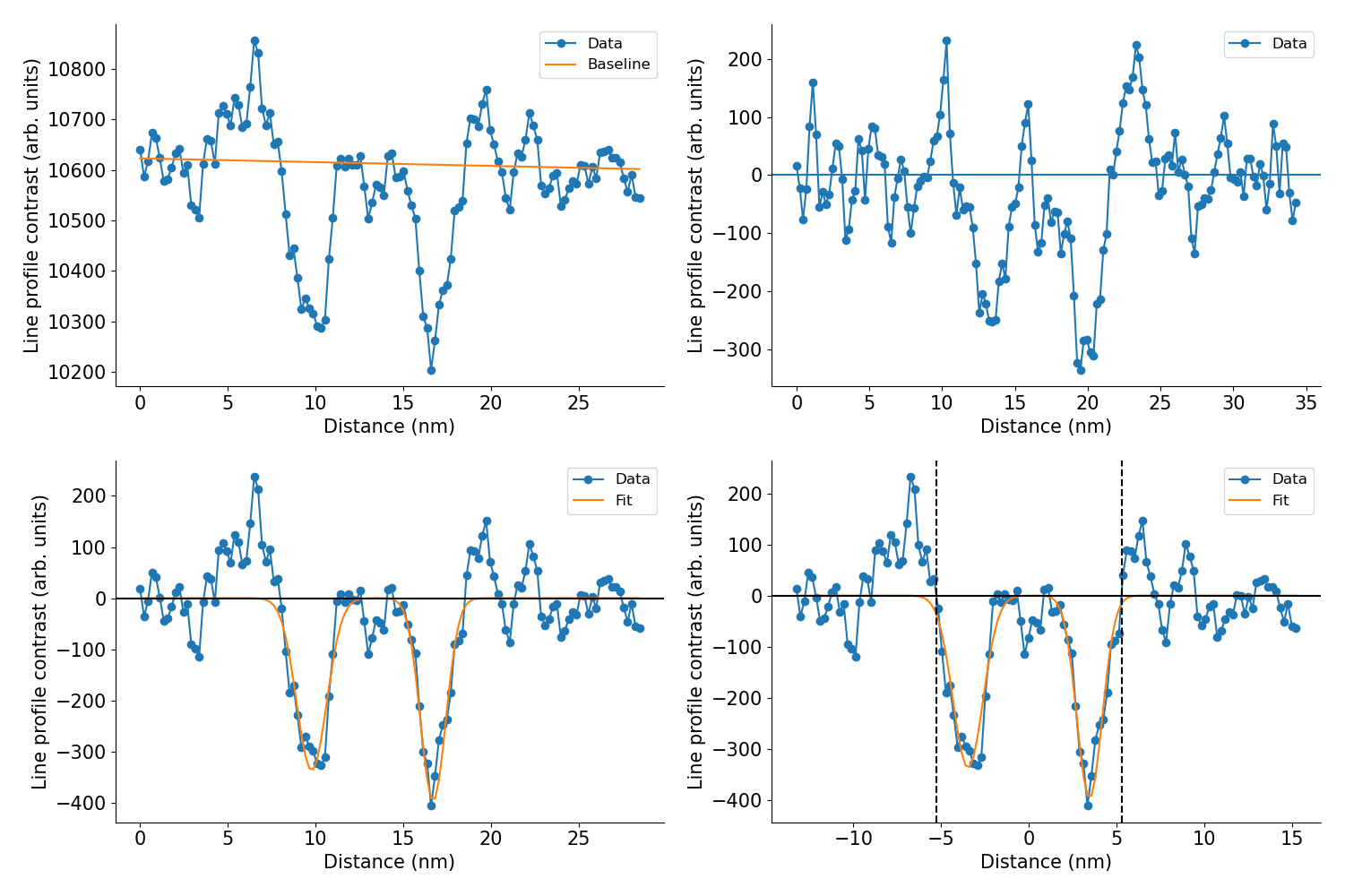


**Figure S11.** Data processing of a representative line profile of flash diluted NTs. A. Modelling the baseline using a linear function. B. Subtracting the baseline from the line profile. C. Fitting the line profile with two Gaussian functions. D. Centering the line profile between the two inner dips. The intersection of the line profile with the baseline are shown by dashed lines.

V. Length of NTs after flash-dilution process

Fig. S12 shows low-magnification cryo-TEM images of DWNTs (A, 50000x), and flash diluted NTs (B, 29000x). There is no observable change in the length of NTs after flash-dilution considering the sight of view of the cryo-TEM grid. Fig. S12 A and S12 B are low magnification overview pictures taken after the high resolution pictures and experienced at least 100 e/Å^2^. There is no radiation damage to the NTs themselves, but only at the holey carbon support which is due to the contamination from the manufacturing process of the quantifoil.


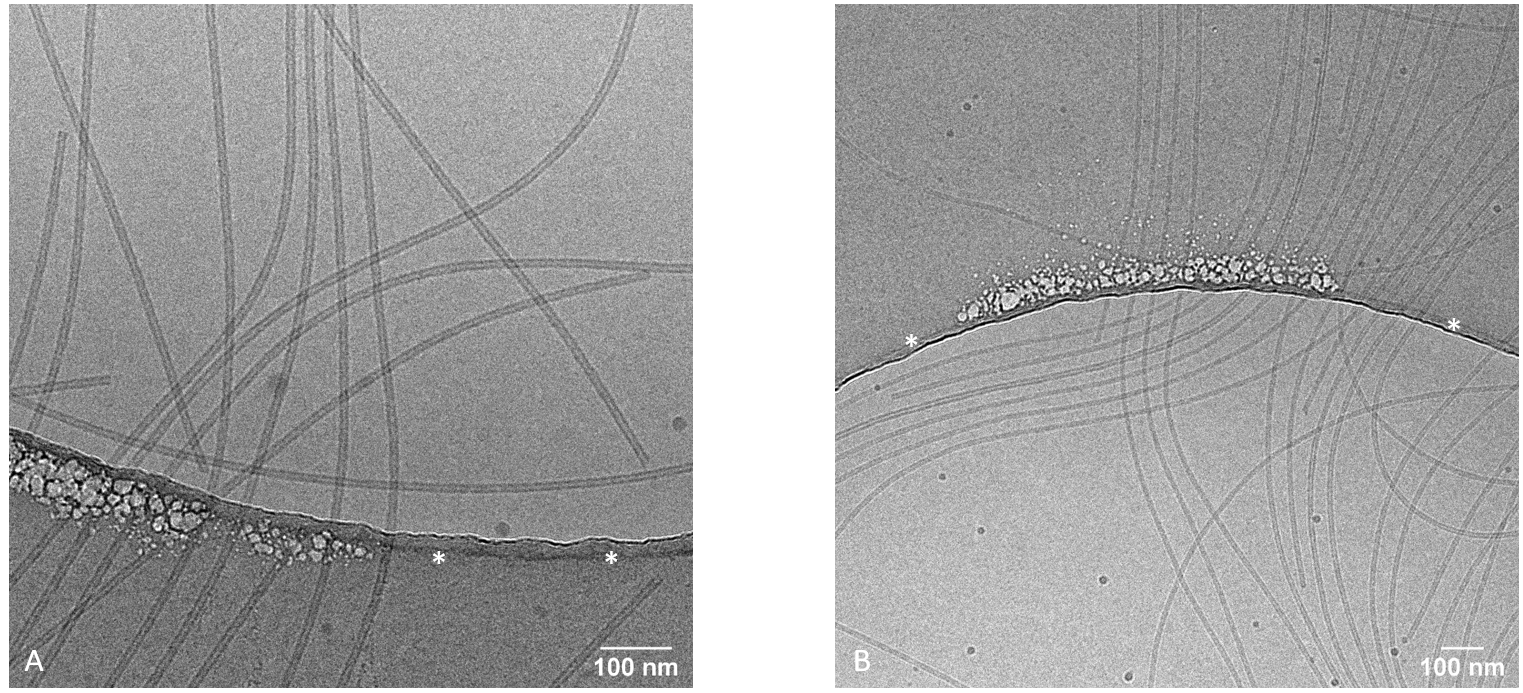


**Figure S12.** Low magnification cryo-TEM image of DWNTs (A, 50000x) and flash diluted NTs (B, 29000x). There is no shortening of NTs after the flash-dilution process considering the field of view of the cryo-TEM grid (which outline is marked by *).

VI. Molecular model building and MD simulation

MD simulations were performed using the Gromacs 2018 simulation package^5^ with the GAFF force field^6^ in combination with the TIP3P water model^7^. Before starting the production phase, the system was minimised with the steepest descent algorithm for 1000 steps and equilibrated in the NVT and NPT ensembles for 1 ps each. The production phase took place in the NPT ensemble for 1 ns. In both NPT simulations, the pressure was kept constant by using the Berendsen barostat^8^ in an isotropic pressure bath with a time constant of 1 ps and compressibility of 4.5 10^-5^ bar^-1^. In all simulations, the temperature was kept constant at 300 K with the V-rescale algorithm^9^ and a coupling constant of 0.1 ps. For the short range interactions (electrostatic and van der Waals), the Verlet cut-off scheme was used with a 1.4 nm cut-off. The long range interactions were calculated with the reaction field method^10^ and the dielectric constant of the surrounding medium was set to 54. The integration time step of the equations of motion was 2 fs during the equilibration and production phase.

During the equilibration, the aromatic core of the C8S3 molecules in the inner and outer wall was restrained with harmonic potentials (force constant of 1000 kJ mol^-1^ nm^-2^) to allow the solvent and side chains to relax and adopt more natural conformations. Harmonic restraints were still applied to the inner wall to prevent the exposed inner cylinder from collapsing and allow a fair comparison of the top and bottom inner wall of the structure. Furthermore, the system was translated and rotated to remove the center of mass motion of the nanotube every 100 steps in order to keep the structure in the center of the box.

VII. Projected nuclear charge density and TEM image calculation with abTEM

A jupyter notebook was developed based on the examples given in the walkthrough on the github page of the program suite abTEM (https://github.com/jacobjma/). The atomic coordinates resulting from MD simulations (see section on molecular model) were imported using the MDAnalysis tool^11^ (https://www.mdanalysis.org/) and used to build an abTEM Atoms object.

The projected nuclear charge density image (including water as the solvent) was created by first making a histogram of each atom type present in the system (H, C, N, O, Na, S, Cl), multiplying each histogram by the charge of the nucleus (1, 6, 7, 8, 11, 16, 17) and adding these; the resulting distribution was rendered using matplotlib (Fig. S13). Line profiles of the projected nuclear charge density across the sample were obtained by averaging, smoothing, and scaling individual line profiles taken from regions with the SW and DW sections of the tube (Figs. 5C, 5D (main text) and Fig. S8).


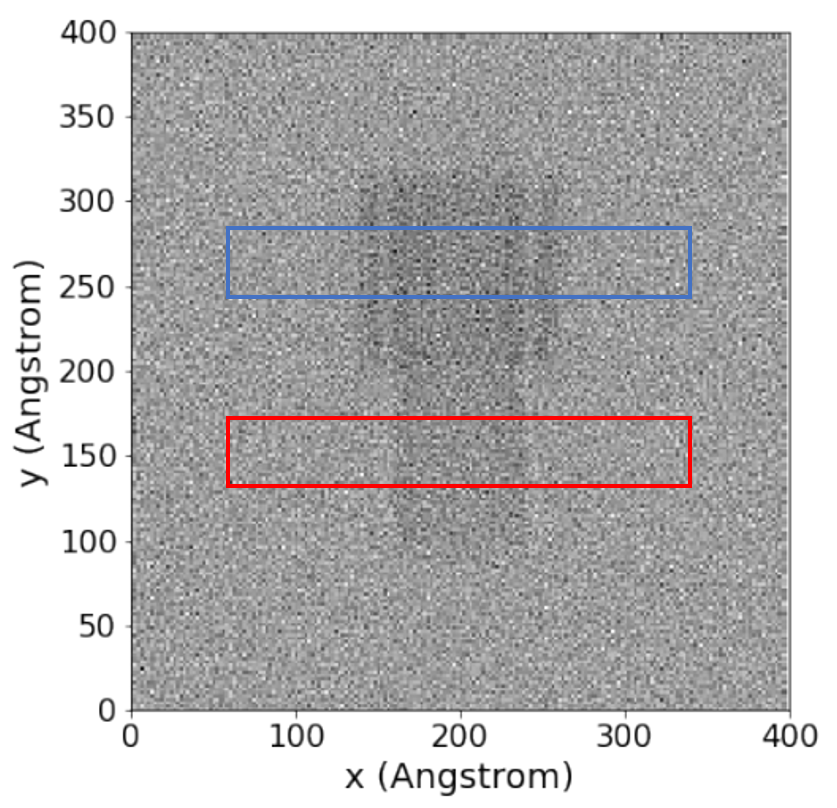


**Figure S13.** Projected nuclear density image of DW and SW sections, which are highlighted by blue and red rectangles, respectively. Here, water is considered as the solvent.

For calculation of the HRTEM images, first, appropriate atom numbers were supplied for all atoms in the abTEM Atoms object. Then, the exit wave of a plane wave is calculated by constructing a representation of the potential through which the plane wave is propagated, using the following settings:

*sampling* = 1.0,

*slice_thickness* = 0.1 Angstrom,

*parametrisation* = 'lobato' ^12^,

*projection* = 'finite',

*z_periodic* = False

The plane wave was constructed with an energy of 200 keV and with a real space resolution of 1.0 Angstrom and numerically propagated across the electrostatic potential with the multislice algorithm^13^ (as can be seen above, the slice thickness is 0.1 Å. The total sample thickness is approximately 40 nm, or 400 Angstrom). The exit wave was subsequently converted to an HRTEM image by applying a Contrast Transfer Function (CTF). CTF settings were taken close to those of the electron microscope used in the experiment, being:

*energy* = wave.energy, # 200,00 eV

*semiangle_cutoff* = sa, # approx. 9.3 mrad, calculated from wave.energy and Cs.

*focal_spread* = 100, # 100 Å, minimum calculated from Cs and spread in acceleration voltage.

*defocus* = df, # Å, varied

*Cs* = -2e-3 * 1e10, # 2e07 Å, spherical aberration, 2 mm converted to Å

*angular_spread* = 0.1 Angstrom,

*gaussian_spread* = 0 Angstrom,

Contrast profiles for the SW and DW sections (Figs. 5C and 5D in the main text) were generated from the image by averaging over 5 line profiles, each with a length of 20 nm, across the tube in each of the SW and DW sections. The averaged profiles were further smoothened by taking a rolling average over 7 points.

**Supplementary Information References**

(1) Clark, K. A.; Krueger, E. L.; Vanden Bout, D. A. Temperature-Dependent Exciton Properties of Two Cylindrical J-Aggregates. *J. Phys. Chem. C* **2014**, *118* (42), 24325–24334. https://doi.org/10.1021/jp507791q.

(2) Von Berlepsch, H.; Kirstein, S.; Hania, R.; Pugžlys, A.; Böttcher, C. Modification of the Nanoscale Structure of the J-Aggregate of a Sulfonate-Substituted Amphiphilic Carbocyanine Dye through Incorporation of Surface-Active Additives. *J. Phys. Chem. B* **2007**, *111* (7), 1701–1711. https://doi.org/10.1021/jp065826n.

(3) Eisele, D. M.; Arias, D. H.; Fu, X.; Bloemsma, E. A.; Steiner, C. P.; Jensen, R. A.; Rebentrost, P.; Eisele, H.; Tokmakoff, A.; Lloyd, S.; Nelson, K. A.; Nicastro, D.; Knoester, J.; Bawendi, M. G. Robust Excitons Inhabit Soft Supramolecular Nanotubes. *Proc. Natl. Acad. Sci. U. S. A.* **2014**, *111* (33), E3367–E3375. https://doi.org/10.1073/pnas.1408342111.

(4) Kriete, B.; Feenstra, C. J.; Pshenichnikov, M. S. Microfluidic Out-of-Equilibrium Control of Molecular Nanotubes. *Phys. Chem. Chem. Phys.* **2020**, *22* (18), 10179–10188. https://doi.org/10.1039/d0cp01734e.

(5) Abraham, M. J.; Murtola, T.; Schulz, R.; Páll, S.; Smith, J. C.; Hess, B.; Lindah, E. Gromacs: High Performance Molecular Simulations through Multi-Level Parallelism from Laptops to Supercomputers. *SoftwareX* **2015**, *1*–*2*, 19–25. https://doi.org/10.1016/j.softx.2015.06.001.

(6) Wang, J.; Wolf, R. M.; Caldwell, J. W.; Kollman, P. A.; Case, D. A. Development and Testing of a General Amber Force Field. *J. Comput. Chem.* **2004**, *25* (9), 1157–1174. https://doi.org/10.1002/jcc.20035.

(7) Jorgensen, W. L.; Chandrasekhar, J.; Madura, J. D.; Impey, R. W.; Klein, M. L. Comparison of Simple Potential Functions for Simulating Liquid Water. *J. Chem. Phys.* **1983**, *79* (2), 926–935. https://doi.org/10.1063/1.445869.

(8) Berendsen, H. J. C.; van der Spoel, D.; van Drunen, R. GROMACS: A Message-Passing Parallel Molecular Dynamics Implementation. *Comput. Phys. Commun.* **1995**, *91* (1–3), 43–56. https://doi.org/10.1016/0010-4655(95)00042-E.

(9) Bussi, G.; Donadio, D.; Parrinello, M. Canonical Sampling through Velocity Rescaling. *J. Chem. Phys.* **2007**, *126* (1), 014101-1 - 014101-7. https://doi.org/10.1063/1.2408420.

(10) Tironi, I. G.; Sperb, R.; Smith, P. E.; Van Gunsteren, W. F. A Generalized Reaction Field Method for Molecular Dynamics Simulations. *J. Chem. Phys.* **1995**, *102* (13), 5451–5459. https://doi.org/10.1063/1.469273.

(11) Michaud-Agrawal, N.; Denning, E. J.; Woolf, T. B.; Beckstein, O. MDAnalysis: A Toolkit for the Analysis of Molecular Dynamics Simulations. *J. Comput. Chem.* **2011**, *32* (10), 2319–2327. https://doi.org/10.1002/jcc.21787.

(12) Lobato, I.; Van Dyck, D. An Accurate Parameterization for Scattering Factors, Electron Densities and Electrostatic Potentials for Neutral Atoms That Obey All Physical Constraints. *Acta Crystallogr. Sect. A Found. Adv.* **2014**, *70* (2014), 636–649. https://doi.org/10.1107/S205327331401643X.

(13) Cowley, J. M.; Moodie, A. F. The Scattering of Electrons by Atoms and Crystals. I. A New Theoretical Approach. *Acta Crystallogr.* **1957**, *10* (10), 609–619. https://doi.org/10.1107/S0365110X57002194.
